# Fitting Early Phases of the COVID-19 Outbreak: A Comparison of the Performances of Used Models

**DOI:** 10.3390/healthcare11162363

**Published:** 2023-08-21

**Authors:** Veronica Sciannameo, Danila Azzolina, Corrado Lanera, Aslihan Şentürk Acar, Maria Assunta Corciulo, Rosanna Irene Comoretto, Paola Berchialla, Dario Gregori

**Affiliations:** 1 Unit of Biostatistics, Epidemiology and Public Health, Department of Cardiac, Thoracic, Vascular Sciences, and Public Health, University of Padova, 35131 Padova, Italy; veronica.sciannameo@unito.it (V.S.); danila.azzolina@unife.it (D.A.); corrado.lanera@unipd.it (C.L.); maria.corciulo@ubep.unipd.it (M.A.C.); rosannairene.comoretto@unito.it (R.I.C.); 2 Center of Biostatistics, Epidemiology and Public Health, Department of Clinical and Biological Sciences, University of Torino, 10124 Turin, Italy; paola.berchialla@unito.it; 3 Department of Environmental and Preventive Sciences, University of Ferrara, 44121 Ferrara, Italy; 4 Department of Actuarial Sciences, Hacettepe University, 06230 Ankara, Turkey; aslihansenturkacar@gmail.com; 5 Department of Public Health and Pediatrics, University of Torino, 10124 Turin, Italy

**Keywords:** COVID-19, prediction model, early phase, epidemic models, prevention policy

## Abstract

The COVID-19 outbreak involved a spread of prediction efforts, especially in the early pandemic phase. A better understanding of the epidemiological implications of the different models seems crucial for tailoring prevention policies. This study aims to explore the concordance and discrepancies in outbreak prediction produced by models implemented and used in the first wave of the epidemic. To evaluate the performance of the model, an analysis was carried out on Italian pandemic data from February 24, 2020. The epidemic models were fitted to data collected at 20, 30, 40, 50, 60, 70, 80, 90, and 98 days (the entire time series). At each time step, we made predictions until May 31, 2020. The Mean Absolute Error (*MAE*) and the Mean Absolute Percentage Error (*MAPE*) were calculated. The GAM model is the most suitable parameterization for predicting the number of new cases; exponential or Poisson models help predict the cumulative number of cases. When the goal is to predict the epidemic peak, GAM, ARIMA, or Bayesian models are preferable. However, the prediction of the pandemic peak could be made carefully during the early stages of the epidemic because the forecast is affected by high uncertainty and may very likely produce the wrong results.

## 1. Introduction

COVID-19 is a global-scale pandemic infectious disease caused by the SARS-CoV-2 virus, which emerged in November 2019 in Wuhan (Hubei), China. On January 29, 2020, the first two cases were registered in Italy. On January 1, 2022, the official Italian statistics on COVID-19 declared 6,266,939 confirmed cases and 137,513 deaths [1].

Since the early stages of the outbreak, modeling the epidemic growth pattern has been considered crucial for understanding the evolution of the contagion. This research compares the efficacy of different modeling approaches during the early, uncertain phase of the pandemic, shedding light on factors that drive accurate predictions and discrepancies.

Forecasting the outbreak peak and estimating the number of R0 disease reproductions was crucial to guiding the implementation of prevention and control measures [2]. Several predictive and forecasting models have been proposed in the literature, especially during the early phases of the pandemic’s evolution, by different organizations, such as research institutes, academic organizations, and consulting companies [3]. These models have been developed with the primary objective of supporting health systems in the formulation of appropriate prevention or containment policies [4]. 

Among the proposed models, the most traditional infectious disease prediction tools include differential equations and time series. Differential equation models reflect the dynamics of infectious diseases by describing the relationship between the rate of change at fixed times and the number of individuals in different compartments of the population. Currently, the most widely applied compartmental models are the Susceptible-Infected-Recovered (SIR) model, proposed by Kermak and McKendrick in 1927 [5], the Susceptible-Infected-Recovered-Death (SIRD) model, and the Susceptible-Exposed-Infected-Recovered (SEIR) model. These models were widely applied to predict the evolution of the outbreak of many epidemics, such as Ebola or SARS, showing good prediction performance [2,6,7]. However, compartmental models are strongly parameter-dependent and can be extremely sensitive to small changes in assumed scenarios [8]. Furthermore, in several cases, they ignore variations in transmission parameters in the different stages of epidemics, even if recent developments have introduced a time-dependent parameterization [9,10]. During the early phases, the suboptimal availability of data, the rapid evolution of the pandemic, and the unprecedented control measures adopted made their use to predict the COVID-19 outbreak challenging. More complex compartmental models such as SEIR, which are more biologically realistic and embed more epidemiological information, require more parameters to be estimated and therefore lead to a higher degree of uncertainty in prediction [11].

Compared to compartmental models based on epidemiological assumptions and equations, there are phenomenological models, which are data-driven and try to fit a trend and predict the outbreak unfolding from it. Data-driven models have the advantage of allowing simple calibrations of the empirical data collected. Among data-driven models, the most popular are the exponential model, the logistic growth model, the generalized logistic growth model, and the Poisson model, which have been widely applied to describe other epidemics and the COVID-19 outbreak [12,13,14,15,16,17,18]. However, these models have several limitations, and they are only applicable when enough data points are available [15].

In some studies [19,20,21], the epidemiological trend of prevalence and incidence of the COVID-19 outbreak is estimated through the ARIMA model, which is composed of the autoregressive (AR), integrated (I), and moving average (MA) models. Time series prediction models are based on random processes and predict the path of infectious disease by analyzing one-dimensional time series of data [12]. However, ARIMA modeling requires a stationary sequence of data, and this is not the case for the COVID-19 time series, consequently requiring a difference or logarithmic transformation of the data sequence. Furthermore, ARIMA models lack accuracy and are not used in the very early stages of the epidemic, as they can provide useful results only when at least 16–20 data points are available [22,23]. Other authors [24,25] applied a more advanced Bayesian methodology, taking advantage of previous information on the evolution of COVID-19 obtained from other nations. This technique could be used due to the comparable disease trajectories between countries in terms of internal timing, even if COVID-19 has spread to different countries at different times. With this approach, even in the early stages of the epidemic, when only a few data points are available, it is possible to obtain good prediction estimates. Finally, machine learning (ML) models such as long-short-term memory (LSTM) or modified autoencoder (*MAE*) were also applied in some studies [26,27], using SARS data for training. However, ML techniques are most effective when a large amount of data is available, and this is usually not the case in the early stages of an outbreak. Furthermore, ML methods are often ‘black boxes,’ which are not inherently interpretable models.

In general, at the early stage of the epidemic, the predictive models for COVID-19 were affected by the rapid evolution of the outbreak, with new data rapidly accruing that made the prediction highly uncertain and changing as new data became available. However, statistical models are meaningful tools that offer crucial insights to policymakers. In this general framework, it is important to characterize the strengths and weaknesses of the epidemic predictive tools as supporting instruments for the planning of the COVID-19 prevention policy [28]. A better understanding of the epidemiological implications of the different statistical approaches seems crucial, as government interventions and control measures in all sectors strongly depend on the prediction of the outbreak [28]. The first wave of the COVID-19 pandemic represents a critical period in the early stages of the pandemic when there was a rapid increase in the number of cases and deaths globally. This period was characterized by many attempts to predict the future trajectory of the pandemic and its potential impact on healthcare systems and economies, despite the limited data and understanding available at the time.

This study aims to explore the concordance and discrepancies of the outbreak predictions provided by considering the most commonly used models considered in the literature to perform predictions during the early stages of the pandemic. By focusing on the first wave of the pandemic, it is possible to capture the range of modeling approaches that were developed and applied during this critical period, providing a comprehensive picture of the state-of-the-art modeling approaches used to predict the spread and impact of the virus [29]. Some efforts have been made in the literature to characterize the epidemic’s evolution, especially during the first stages of the pandemic, especially in China. In a particular example, Wen and colleagues [30] delve into heterogeneous epidemic modeling within enclosed spaces. Their Bayesian estimation approach offers nuanced insights into the progression of diseases in spatially confined settings. While their context is more specific, their methodological rigor provides valuable guidance for predictive tools, especially in regions with high population densities. Moreover, Lin and colleagues [31] present a conceptual model focused on the initial outbreak in Wuhan. Their emphasis on individual reactions and governmental actions aligns with our approach of considering non-pharmaceutical interventions and public response as critical predictive variables. Their findings on Wuhan, the epicenter of the outbreak, offer useful insights into the early stages of the pandemic.

However, to our knowledge, this research represents a first effort to provide insights into the strengths and weaknesses of different modeling approaches in the face of an emerging, rapidly evolving, and uncertain situation. The study can help identify the factors that contribute to accurate predictions and those that lead to discrepancies. The Italian scenario has been considered for this research because this country played a pivotal role during the early stages of the COVID-19 pandemic, providing researchers, policymakers, and the global community with essential insights into the virus’s spread, impact, and potential mitigation strategies. The epidemic situation during the early stages of the pandemic, combined with the richness of Italian data and the varied policy responses across its regions [32], made Italy an ideal choice for a comprehensive comparison of COVID-19 predictive models [33]. Italy was the first European country to be severely impacted by COVID-19; the rapid surge in cases and the mortality excess in 2020, directly and indirectly, related to the virus [34], served as an early indication of the potential scale and severity of the pandemic in the Western world [13]. Moreover, the Italian health authorities provided regular, detailed updates, making it a valuable dataset for predictive modeling during the early stages of the pandemic [35].

Such a study could be particularly useful for informing future modeling efforts and improving our ability to predict the spread and impact of emerging infectious diseases. It could also help policymakers and public health experts better understand the limitations of modeling approaches and the importance of incorporating new data and knowledge as it becomes available. 

To achieve this objective, we applied compartmental and data-driven models to forecast the number of COVID-19 infections in Italy as they evolved during the first wave and compared their predictions.

## 2. Materials and Methods

### 2.1. Data

Models were implemented based on the total number of confirmed cases reported by the Italian Civil Protection Department [1], which is a cumulative number, and the number of new daily positive cases (i.e., the difference between two consecutive days of confirmed cases).

The analysis was limited to the first three months of the epidemic, from 24 February to 31 May 2020. Epidemic models were fitted at 20, 30, 40, 50, 60, 70, 80, 90, and 98 days (the entire time series), and at each time step, forecasts were made until day 98 (31 May 2020) to have predictions on timescales ranging from two months to one week.

Data used for analysis are available on the COVID19ita platform [36], which is a web-based tool that reports and describes statistics developed on official sources of Italian Civil Protection pandemic data (https://github.com/pcm-dpc/COVID-19 (accessed on 1 June 2023).

### 2.2. Epidemic Models

We identified statistical models widely applied for predictions during the first epidemic wave of COVID-19 around the world. The statistical approaches selected along with the corresponding reference(s) are summarized in Table 1.

### 2.3. Compartmental Models

The compartmental SIR and SIRD models are mathematical models used to study the spread of infectious diseases in a population. The models assume that individuals can be classified into different compartments based on their disease status and that the transitions between compartments can be described using differential equations. These models can provide valuable insights into the dynamics of an outbreak and inform public health policies and resource allocation.

A SIR model, for example, allocates each person in the population to one of the following compartments: susceptible, infected, or recovered; the SIRD model instead considers the deceased. According to the model, individuals can flow between different compartments. The flows and interaction rates between compartments are known as the “model parameters” [42]. Prior assumptions on these parameters are necessary to model the epidemic growth trend [43].

As the transmission rate, recovery rate, and death rate in Italy during the first wave of the pandemic were still highly debated, the parameters for these models were estimated directly from the data using a nonlinear minimization procedure [44]. In the model implementation, we allowed for the parameters to change over time to account for variations in the epidemic dynamic (e.g., a different transmission rate before and after the implementation of physical distancing and control measures). Other details concerning the models are reported in the Supplementary Materials.

### 2.4. Data-Driven Models

Data-driven models are attractive, especially with the little information available on the evolution pattern of the pandemic, because they do not assume preliminary knowledge of the mechanism of transmission of the disease [45]. In the first wave of the epidemic, the models employed were: (i) exponential models; (ii) quadratic regression models; (iii) logistic regression, generalized logistic regression, and Richards regressions; (iv) Bertalanffy and Gompertz models; (v) generalized additive models (GAM); (vi) Poisson generalized linear model (GLM); (vii) ARIMA-class models; and (viii) empirical Bayesian time series class models.

### 2.5. Model Evaluation

The goodness of fit of the estimated models was evaluated using the mean absolute error (*MAE*), computed as:

$$MAE=\frac{\sum_{t=1}^{n}\;(y_{t}-{\hat{y}}_{t})}{n}$$

where $y_{t}$ is the observed value on a $t^{th}$ day and ${\hat{y}}_{t}$ is the predicted value for the same day. The lower the coefficient, the more accurate the prediction will be [12].

This provides an average of the absolute differences between the forecasted and actual values. It gives a linear penalty for forecasting errors.

The mean absolute percentage error (*MAPE*) [46] was also estimated. *MAPE* is a measure of prediction accuracy and is defined by the following formula:

$$MAPE=\frac{1}{n}\sum_{t=1}^{n}\frac{y_{t}-{\hat{y}}_{t}}{y_{t}}$$

This metric provides an understanding of the error in percentage terms, making it more interpretable, especially when comparing across different scales. 

While *MAE* provides a linear penalty, it might not adequately penalize large errors, especially when the magnitude of the data values varies significantly [47]. The *MAPE* metric, instead, has limitations, especially when actual values are close to zero; this issue can lead to very large percentage errors. It is also sensitive to outliers and can sometimes provide an overoptimistic view of the model’s performance [47]. No single metric can capture the comprehensive performance of a model; for this reason, this research used a combination of *MAE* and *MAPE*.

The *MAE* is the most widely used measure of forecast error in time series analysis [48]. *MAE* is reported as an alternative measure to *MAPE*. The *MAPE* is usually chosen over the classical R^2^ because it does not depend on the unit of measurement and represents an error metric defined in percentage values. Furthermore, for interpretability reasons, it is often referred to as quantifying the error in percentage rather than in quadratic terms [47]. All statistical analyses were performed with R version 3.6.1 [49].

## 3. Results

The *MAE* of the models is reported in the Supplementary Materials, along with the *MAPE* prediction accuracy measure reported in Tables S2 and S3 in the Supplementary Materials. The prediction precision is computed based on the forecast of the number of cases until 31 May 2020. 

### 3.1. Total Cases Fit

**Figure 1:**
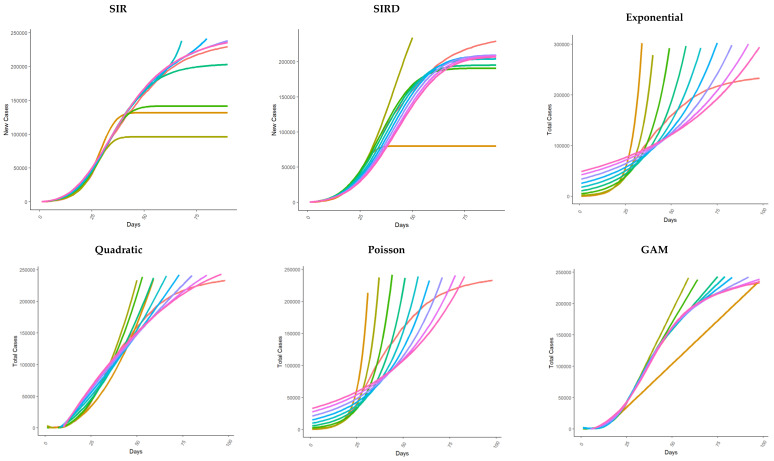

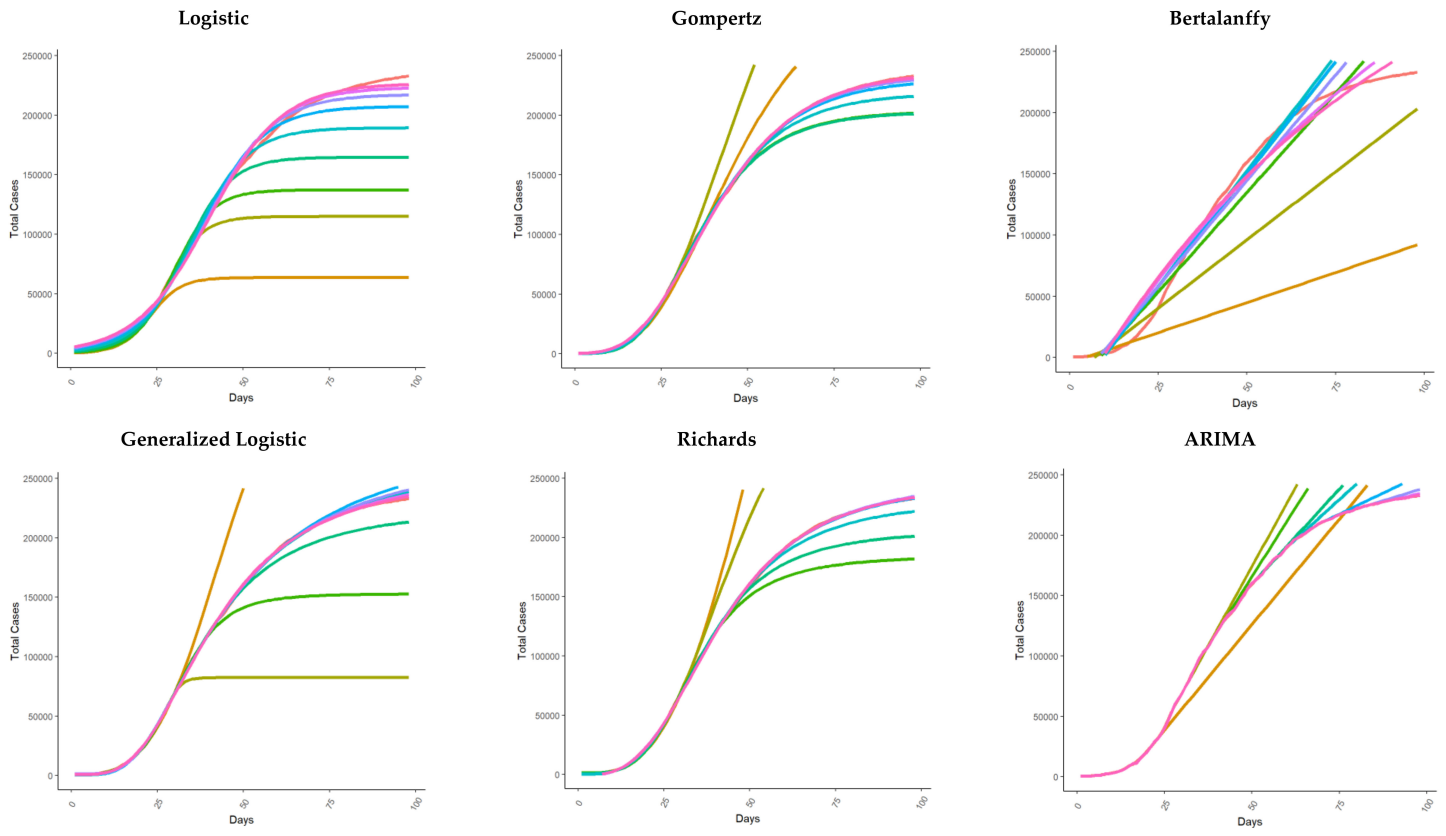

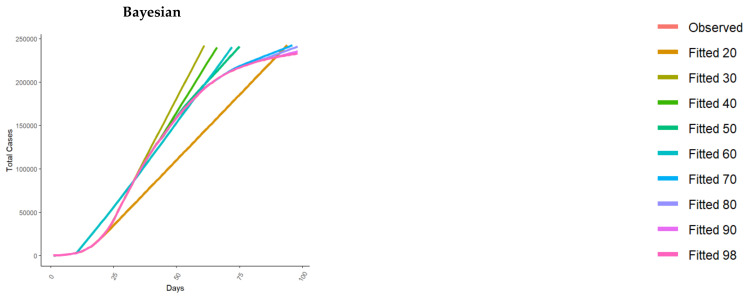
Several models were fitted using the time series of the number of total cases on the first 20, 30, 40, 50, 60, 70, 80, 90, and 98 days from the beginning of the epidemics (February 24, 2020). The model indicated with fitted 98 is the fit for the whole time series from February 24 to May 31. Each model was used to predict the total number of total cases up to the 98th day (May 31, 2020).

The compartmental SEIR and SIRD models misspecified the total number of cases during the overall forecast window, especially considering the estimate of the first 30 days of the pandemic (Figure 1).

The *MAE* estimate is greater than 40,000, considering several time windows for the estimation, especially for the SIR model (Supplementary Materials Table S2). The Poisson and exponential models exhibit particularly high absolute error values (*MAE*) on average. The estimates of the exponential model, in particular, are out of control for the entire epidemic period considered. The return estimates of the quadratic model are less biased in terms of *MAE* and *MAPE* on the 90th day of the epidemic (Supplementary Materials Table S2). 

Regarding logistic-derived models (i.e., Gompertz, Generalized Logistic, Bertanlaffy, Richards), in the first 20 days, Bertanlaffy’s model performs better than the others (*MAE* < 10). In the course of the pandemic period, the performances of all these models remained similar (the *MAPE* is reported in Supplementary Materials, Table S2).

Models based on smoothing functions performed very well on observed data in both frequentist (GAM) and Bayesian settings (Figure 1). *MAE* and *MAPE* are particularly contained for these parametrizations on the 70th day of the epidemic. 

The time-series ARIMA models perform better than the polynomial models (Figure 1), with lower observed *MAE* and *MAPE* until day 70. Furthermore, considering the last 20 days of the epidemic, a Bayesian estimation approach minimizes both *MAE* and *MAPE* compared to the other methods (Supplementary Materials Table S2).

### 3.2. New Cases Fit

**Figure 2:**
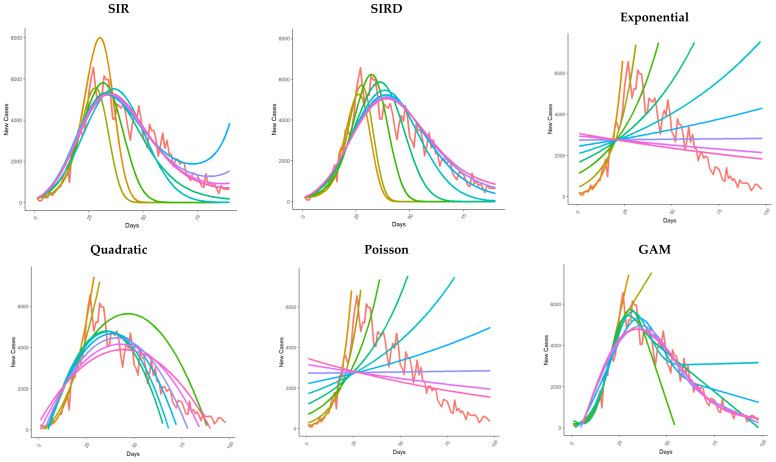

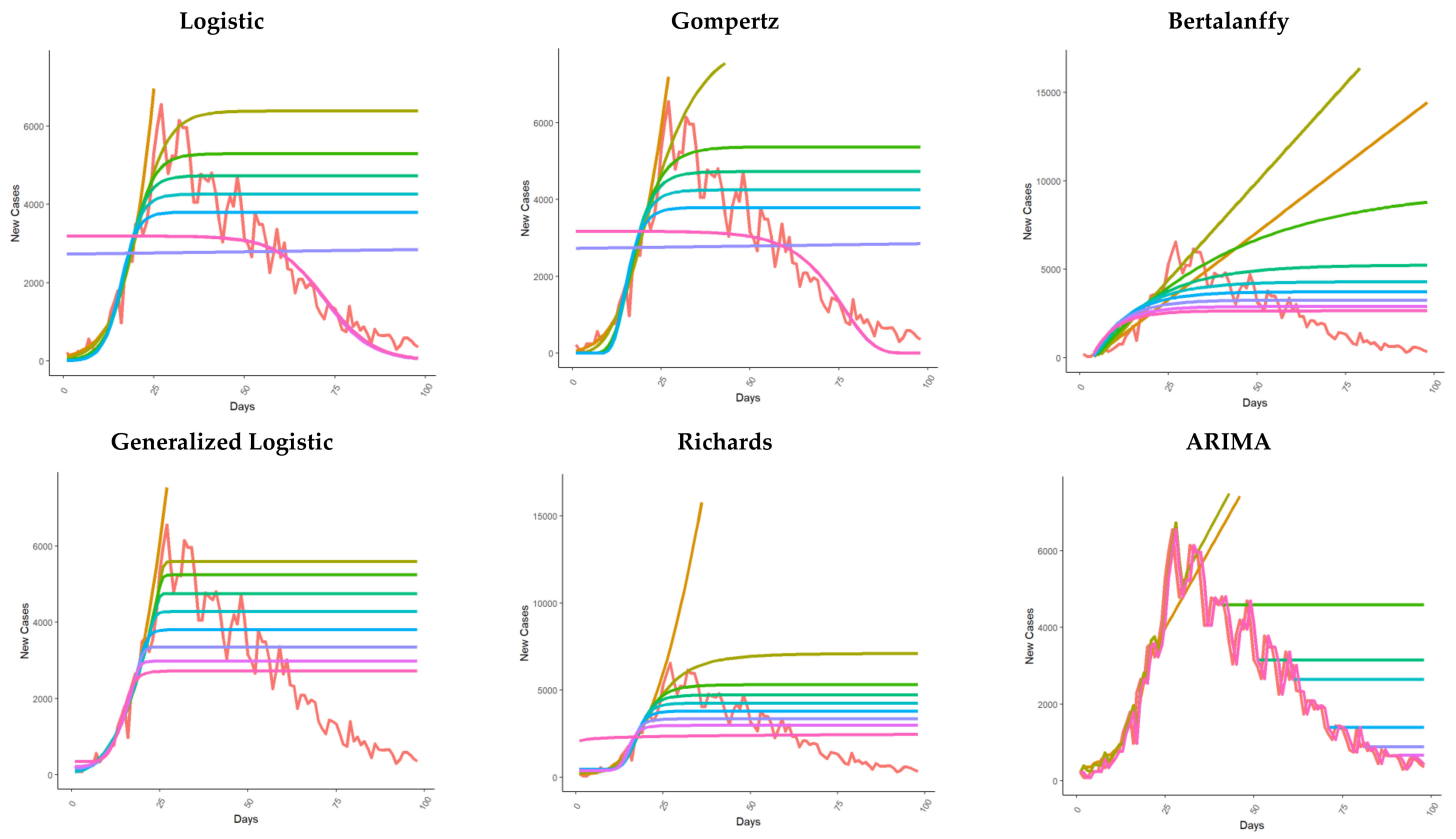

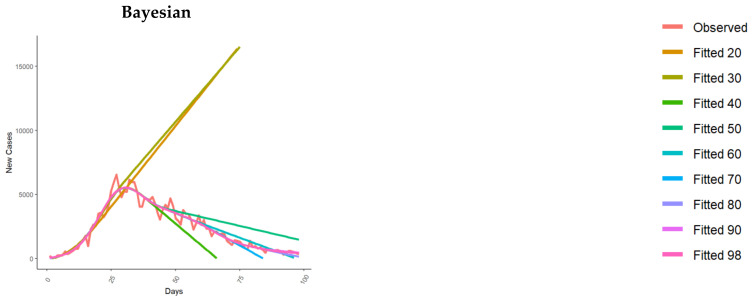
Epidemic models were fitted using the number of new daily cases reported on the first 20, 30, 40, 50, 60, 70, 80, 90, and 98 days of the epidemics. The models were used to predict the number of new daily cases of COVID-19 up to May 31. The model indicated with fitted 98 is the fit for the whole time series from February 24 to May 31.

The compartmental SEIR and SIRD models overestimated the number of new cases, especially during the first 20 days of the epidemic (Figure 2). However, the *MAPE* estimate remains lower than one during all periods considered for both compartmental models (Supplementary Materials Table S3).

Moreover, in this context, for the classical Poisson and exponential models, particularly high absolute error values (*MAE*) on average have been observed. The estimates of these models are still completely biased for the whole epidemic period considered. The quadratic model return estimates are less biased in terms of *MAE* and *MAPE* in comparison with the other classical Poisson and exponential models (Supplementary Materials Table S3). The performances of logistic-derived models remain similar to each other throughout the period considered. However, during the last 20 days, the *MAPE* achieved values greater than one, indicating a possible lack of fit for these models after reaching the pandemic peak. This pattern has been observed, especially using the Logistic, Gompertz, and Richards models (Supplementary Materials Table S3). Regarding the total case fit, models based on smoothing functions performed very well on observed data in both a frequentist (GAM) and Bayesian setting (Figure 2), with both *MAE* and *MAPE* particularly contained during all considered periods. For time-series ARIMA models, *MAE* and *MAPE* have been reported to be less than one during all the considered time frames, suggesting good model performance. Once again, a Bayesian approach minimizes both *MAE* and *MAPE* compared to the other methods considered for this analysis in the last 20 days (Supplementary Materials Table S3). 

## 4. Discussion

Modeling the epidemic growth pattern is essential to predicting the evolution of the pandemic, which is a fundamental issue for the implementation of control and prevention measures [2]. Predictive models allow an early evaluation of the behavior of the pandemic by estimating its trend, peaks, and decline in new cases (incidence) [50]. This information is useful to forecast the demand for acute medical services, determine the timeframes for containment measures, and plan the need for healthcare providers and the resources for prevention and treatment, such as personal protective equipment (PPE), ventilators, etc. [51] and the proper allocation of human resources in emergency settings [52].

Several models have been extensively applied to predict the growth pattern of the COVID-19 epidemic; however, as we pointed out in this study, different approaches lead to dissimilar results [53]. Consequently, it is necessary to better understand the implications of these different approaches and to determine which model could be more appropriate at a specific stage of the epidemic, considering the epidemiological purposes of the analyses.

Compartmental models have been extensively used for different infectious diseases, for example, measles, dengue fever, influenza, HIV, the 2002 SARS epidemic, and the Ebola disease [54,55]. These models are strongly parameter-dependent, and their purpose is to outline the general behaviors of the epidemic series while predicting several variables at the same time. One of the advantages of these approaches is that they are not very demanding in terms of the number of input parameters to be implemented; however, some basic knowledge about disease transmission mechanisms should be assumed [56]. For these reasons, while they can be very reliable when outbreak behaviors are known, they can lead to misleading or even wrong predictions when not enough information is available [56], as we observed using compartmental models to predict the unfolding of the pandemic during its early stages. On the contrary, nowadays, mechanisms of virus transmission are known; compartmental models are appealing for estimating the long-term effects of the vaccination campaign, for example, and the transmission mechanisms of new virus variants [57,58]. Moreover, other parametrizations of the SIR models have recently been reported in the literature; for example, iterative algorithms for approximating the incidence variable, which allows for the estimation of the model parameters from the number of observed cases, have been developed [59]. The SIR model has also recently been related to a parametric Gompertz distribution for the infected cases [60].

In our analysis, the data-driven models’ GAM, ARIMA, and Bayesian methods revealed the most suitable performance in the prediction of the new confirmed cases of COVID-19. These approaches are more flexible and allow modeling of the descending phase of the epidemic trend. A considerably lower *MAPE* is associated with GAM, ARIMA, and Bayesian models compared to other approaches. More in detail, the use of GAM [61] is preferable in the early stages of the pandemic, while ARIMA and Bayesian models gave better predictions in the final stages, as already reported in the literature [22]. The precision and ability of time series models to rapidly adapt to disease changes by providing performant short-term forecasts have also been demonstrated in other recent research on the COVID-19 pandemic [62].

Among the classical models, the quadratic model has inaccuracies both in the initial phase, as it may not be able to predict the interruption of the exponential trend, and in the subsequent one, as a quadratic trend seems to predict an epidemic peak too quickly [39]. In contrast, the exponential family models cannot capture the descending trend after the peak is reached, giving overestimated predictions. Furthermore, for the forecast of the cumulative number of confirmed cases of COVID-19, the exponential and Poisson models can only be applied in the early stages of the epidemic [16]; however, they have a considerably higher *MAPE* compared to the other models.

Models of the logistic family (logistic, Gompertz, generalized logistic, and Richards model) are more suitable for predicting when the plateau of cumulative epidemic growth is reached. More in detail, the Gompertz model is the best-performing one due to its flexibility in dealing with an asymmetry in the logistic S-shape, followed by the Richards model. These flexible variants of the logistic model have also been applied in other studies on the first phases of the pandemic in Italy [63,64]. The logistic model and the generalized logistic model perform well only when enough data points are accumulated, as also demonstrated elsewhere [65]. 

The lesson learned from this comparative analysis is the absence of a unique model that best predicts epidemic patterns. The choice of the most suitable parameterization depends on the epidemiological objectives of the analysis and the level of knowledge of the disease and its transmission mechanisms. Certainly, under uncertain conditions, sensitivity analyses on parameters and assumptions are useful [66]. The accuracy of the predictions depends on various factors, including the availability and quality of data, the modeling approach, and the complexity of the pandemic dynamics. In this scenario, the early stages of the COVID-19 pandemic were characterized by many unknowns and uncertainties, including limited data on the virus and its transmission mechanisms as well as the effectiveness of various interventions. As a result, it may have been challenging to make highly accurate predictions, especially given the limitations of available data and knowledge at the time [67]. Concerning the quality of the data, in Italy, as in many countries worldwide, there were concerns about the accuracy of COVID-19 diagnosis, particularly during the early phases of the pandemic. The initial lack of established testing protocols and potential shortages in testing kits might have led to both over- and under-reporting [68]. Over time, however, with increased testing capabilities and improved diagnostic methodologies, the accuracy of COVID-19 diagnosis likely improved [69].

Moreover, while there were reports in the US about potential financial incentives skewing hospital classifications of COVID-19 patients [70], the situation in Italy appears distinct. The Italian healthcare system is predominantly public and centralized, thereby reducing financial motivations for potential misreporting. However, we cannot entirely rule out systemic biases or administrative errors that might have influenced reporting. Some regions in Italy experienced overwhelming pressure on their healthcare system, which might have inadvertently affected the precision of case recording [13].

To address potential concerns about data integrity, our study utilized data only from official sources such as the Italian Ministry of Health and the Istituto Superiore di Sanità. These agencies have been transparent about their methodologies and have continuously sought to refine their data collection processes as the pandemic unfolded [35].

In the early stages of the epidemic, the purposes of predictive analysis, in general, are to monitor and forecast disease transmission in the short term and plan healthcare policies and interventions [71]. While the study focuses on the early stages of the pandemic and the performance of different prediction models during that period, the findings may still have some applicability to the later stages of the pandemic. For example, the study highlights the importance of considering multiple models and their respective strengths and weaknesses when making predictions during a period of limited data and knowledge. This may still be relevant in the later stages of the pandemic as new variants emerge and vaccination rates increase. Regarding applicability to other cities or regions, the findings may be informative for policymakers and public health officials in other areas facing similar challenges during the early stages of the pandemic. However, it is important to note that the dynamics of the pandemic can vary significantly between regions due to differences in demographics, population density, healthcare systems, and other factors [72]. For this reason, the analyses and results should be updated as soon as new data are available. In uncertain situations, phenomenological modeling approaches are particularly suitable when characterizing the epidemiology of infectious disease in its early phases. These techniques could offer a good starting point for producing early estimates of disease transmission and for generating short-term forecasts of epidemic spread.

### 4.1. Study Limitations

The comparison between models is limited to Italian data on the COVID-19 outbreak in the first epidemic wave. All the models used have their limitations and are more suitable at different stages of the outbreak or when enough data are available. Moreover, many of them are designed for continuous data despite being applied to count data. However, the approaches that we compared are usually applied to forecast time series [73] and were widely used for COVID-19 predictions. 

One potential limitation is that by including the entire time series as a learning dataset, the models may have learned patterns and trends that may not necessarily be representative of the pandemic dynamics going forward. Additionally, by making predictions for the entire period, the models may be overfitting the historical data and not capturing new or emerging trends that could affect future outcomes. However, we included the entire time series as a learning dataset and made predictions for the entire period based on the available data and the goals of the study because this procedure was adopted, given the paucity of data, during the early stages of the pandemic. 

One example was a study by the Imperial College COVID-19 Response Team in the United Kingdom. In this study, the team used a mathematical model to estimate the potential impact of different interventions on the spread of COVID-19 in the UK [74]. By including the entire time series as a learning dataset, the model was able to capture the dynamics of the pandemic in the early stages, when there was limited data and knowledge about the virus. While this approach may have limitations, it was necessary during the early stages of the pandemic when there was limited data and knowledge available. As more data became available and pandemic dynamics evolved, models were able to be updated and refined to improve their accuracy and usefulness for policymakers. Future studies may benefit from using different time windows or incorporating additional data sources to assess the accuracy of pandemic predictions.

### 4.2. Future Research Developments

Other research developments are needed to better investigate the performance of the widely used model in other countries by providing a more comprehensive understanding and collocating our research within a wider landscape, accounting for heterogeneities in prevention policies and potential biases in data reporting and updating.

## 5. Conclusions

Data-driven models are useful to model the early stages of the epidemic. The GAM model is the most suitable for predicting the number of newly confirmed cases; exponential or Poisson models are useful for predicting the cumulative number of cases. When the purpose is to predict the epidemic peak, GAM, ARIMA, or Bayesian models are preferable to detect the number of new cases; meanwhile, Gompertz or Richards are well-performing models in forecasting when the growth plateau is soon to be reached. However, it is necessary to be careful with peak predictions during the early stages of the epidemic, as they will most likely yield the wrong results. Finally, the ARIMA and Bayesian models are more suitable for the detection of new cases in later stages.

## Figures and Tables

**Table 1:** Models considered in the analysis and the corresponding reference(s) of the paper(s) in which each model was applied to COVID-19 data worldwide.

Model	Reference(s)	
SIR	Nesteruk 2020 [37]	Nesteruk et al. predicted the numbers of infected, susceptible, and removed persons versus time. They applied a SIR model to predict the characteristics of the epidemic caused by SARS-CoV-2 in mainland China.
SIRD	Fanelli and Piazza 2020 [38]	Fanelli et al. analyzed the temporal dynamics of the coronavirus disease 2019 outbreak in China, Italy, and France in the time window of 22 January–15 March 2020, via a SIRD model to provide officials with realistic estimates for the time and magnitude of the epidemic peak.
Exponential	Remuzzi and Remuzzi 2020 [13]	Remuzzi et al. observed that in Italy, the number of patients infected since 21 February 2020 closely follows an exponential trend. Assuming the absence of contrast policies and, therefore, if the initial trend remains unchanged, the authors used the data observed up to March 8 to predict the trend of the epidemic curve in the following weeks.
Quadratic Regression	https://www.pangeadds.eu/demos/CoVid19/ (accessed on 1 June 2023) [39]	In [39], the authors applied to the Italian data up to 16 March 2020 a generalization of the exponential model, in which the rate of growth may decrease (or increase) linearly over time. Furthermore, this model is also an approximation for the logistic trend in the proximity of the non-exponential regime.
Logistic regression, Generalized logistic regression, and Richards	Vattay 2020 [14] Wu et al. 2020 [15]	In [14,15], the authors applied logistic regression models to monitor the effectiveness of measures taken by governments in the early phase of the epidemic in Italy and China, respectively, that were able to break the initial exponential trend. Wu et al. [15], using the Chinese experience up to 10 March, analyzed the calibration results also for Japan, South Korea, Iran, Italy, and Europe to make future scenario projections based on the results from different models. In Wu [15], logistic regression, generalized logistic regression, and Richards regression were applied.
Bertalanffy and Gompertz	Jia 2019 [12]	In [12], the authors adopted three kinds of mathematical models, i.e., the logistic model, the Bertalanffy model, and the Gompertz model, to analyze the situation of COVID-19 in China. First, the authors used 2003 SARS data to verify the three models to predict the trend of the epidemic, and then they used the three models to fit and analyze the epidemic trend of COVID-19 in Wuhan and non-Hubei areas in China. They predicted the total number of people expected to be infected, the total death toll, and the end time of the epidemic.
Generalized additive model (GAM)	Izadi and Farzali (2020) [40]	In [40], the authors applied a GAM model to capture the trend of the death rate and predict the occurrence of the peak in Canada.
Poisson generalized linear	Bonetti 2020 [17]	In [17], Bonetti developed a Poisson generalized linear model with a logarithmic link function and polynomial regression on time to report day-by-day estimates of the growth rate of the Italian infected subjects.
ARIMA	Benvenuto et al. 2020 [19]	In [19], the authors proposed the ARIMA model, a simple econometric approach, to predict the trend of the prevalence and incidence of COVID-19. The authors used data up to February 10, extracted from an online interactive dashboard hosted by the Center for Systems Science and Engineering (CSSE) at Johns Hopkins University, Baltimore, USA, created to illustrate the number of confirmed COVID-19 cases, deaths, and recoveries for all affected countries [41].
Empirical Bayesian time series	Liu and Guo 2020 [25]	In [25], Liu and Guo proposed an empirical Bayesian time series framework to predict US cases using data from different countries as a prior reference, using the Johns Hopkins University CSSE data [41]. More in detail, Liu and Guo used the idea of internal time, i.e., the virus spread to different countries at different times, with trajectories different in calendar time but similar in internal time.

## Data Availability

Official data on COVID-19 pandemic are available at https://github.com/pcm-dpc/COVID-19.

## Supplementary Materials

The following supporting information can be downloaded at: https://www.mdpi.com/article/10.3390/healthcare11162363/s1. Table S1. Model parameters description. Table S2. *MAE* and *MAPE* for total cases. Table S3. *MAE* and *MAPE* for new cases. Table S4. Last observed vs last fitted total cases at i-th time. Table S5. Last observed vs last fitted new cases at the i-th time. Refs. [75,76,77,78,79,80,81,82,83,84,85,86,87,88,89] are cited in Supplementary Materials.
