# Supplementary material for: Fitting Early Phases of the COVID-19 Outbreak: A Comparison of the Performances of Used Models
Source: Healthcare (Basel). 2023 Aug 21;11(16):2363. doi: 10.3390/healthcare11162363 (PMC10454512; doi:10.3390/healthcare11162363)
Supplement: Supplementary file 1 [file healthcare-11-02363-s001.zip › healthcare-2522070-supplementary.pdf]

Supplementary Material

Fitting Early Phases of the COVID-19 Outbreak: A Comparison of the Performances of Used Models

Veronica Sciannameo[1,2,+], Danila Azzolina[3,1,+], Corrado Lanera[1], Aslihan Şentürk Acar[4], Maria Assunta Corciulo[1], Rosanna Irene Comoretto[5,1], Paola Berchialla[2++], Dario Gregori[1++*]

+ co-primary

++ co-last

[1]Unit of Biostatistics, Epidemiology and Public Health, Department of Cardiac, Thoracic, Vascular Sciences, and Public Health, University of Padova, Italy

[2]Center of Biostatistics, Epidemiology and Public Health, Department of Clinical and Biological Sciences, University of Torino, Italy

[3]Department of Environmental and Preventive Sciences, University of Ferrara, Ferrara, Italy.

[4]Department of Actuarial Sciences, Hacettepe University, Ankara, Turkey.

[5]Department of Public Health and Pediatrics, University of Turin, Turin, Italy

*** Correspondence:**
Prof. Dario Gregori
Unit of Biostatistics, Epidemiology, and Public Health
Department of Cardiac, Thoracic, Vascular Sciences and Public Health
Via Loredan, 18
35131 Padova – Italy
Phone: +39 049 8275384
Email: dario.gregori@unipd.it

1 Models Considered

1.1 Epidemic models

We identified statistical models widely applied for predictions during the first epidemic wave of COVID-19 around the world. The statistical approaches selected along with the corresponding reference(s) are summarized in Table 1 of the main manuscript; the description of the model parameters is reported in Table S1.

1.2 Compartmental Models

A compartmental model assumes that the overall population is divided into compartments. A SIR model, for example, allocates each person in the population to one of the following compartments: Susceptible, Infected, and Recovered; the SIRD model instead considers also the Deceased. According to the model, individuals can flow between different compartments. The flows and interaction rates between compartments are known as the model parameters (1). Prior assumptions on these parameters are necessary to model the epidemic growth trend (2).
The SIR model assumes that individuals in the population can be classified as susceptible, infected, or recovered. The transitions between these compartments are modeled using a system of ordinary differential equations (ODEs).

Let S(t), I(t), and R(t) be the number of individuals in the population who are susceptible, infected, and recovered at time t, respectively. The basic reproductive number, R0, represents the expected number of secondary cases produced by a typical primary case in a completely susceptible population. It is a key parameter in the model, as it determines whether an outbreak will grow or die out.

The equations for the SIR model are:

$$\mathrm{dS/dt} = -\beta \mathrm{Sl/N}$$
$$\mathrm{dl/dt} = \beta \mathrm{SI/N} - \gamma \mathrm{l}$$
$$\mathrm{dR/dt} = \gamma \mathrm{l}$$

where β is the transmission rate, γ is the recovery rate, and N is the total population size. The first equation represents the rate of change of the susceptible population, which is decreasing as individuals become infected. The second equation represents the rate of change of the infected population, which is increasing due to transmission from the susceptible population and decreasing due to recovery. The third equation represents the rate of change of the recovered population, which is increasing as individuals recover from the disease.

The SIRD model adds a compartment for deaths, represented by the variable D(t). The equations for the SIRD model are:

$$dS/dt = -\beta SI/N$$
$$dl/dt = \beta SI/N - (\gamma + \mu)I$$
$$dR/dt = \gamma l$$
$$dD/dt = \mu l$$

where μ is the mortality rate, representing the proportion of infected individuals who die from the disease. The first three equations are the same as in the SIR model, while the fourth equation represents the rate of change of the deceased population, which is increasing as individuals die from the disease.

As the transmission rate, recovery rate, and death rate in Italy during the first wave of the pandemic were still highly debated, the parameters for these models were estimated directly from the data, using a nonlinear minimization procedure (3). In the model implementation, we allowed for the parameters to change over time, to account for variations in the epidemic dynamic (e.g. a different transmission rate before and after the implementation of physical distancing and control measures).

1.3 Data-Driven Models

Data-driven models are attractive, especially with the little information available on the evolution pattern of the pandemic, because they do not assume preliminary knowledge of the mechanism of transmission of the disease (32). In the following part, a short presentation of the main data-driven models is provided.

1.3.1 Exponential model

The exponential model, which was introduced by Thomas Robert Malthus (4), assumes a geometric growth mechanism for the phenomenon considered. The model is defined by the following equation:

$$y_t = y_0 e^{rt}$$

where y_t is the growth function at a time t, y_0 is the value of y_t when $t = 0$ and r represents the relative increase or decrease of y for a unit increase of time t.
The nonlinear least squares were used for the estimation by considering the standard maximum likelihood (ML) algorithm (5).

1.3.2 Quadratic regression model

The quadratic equation describes an increasing or decreasing trend that changes according to the level of time t (6). The most common parameterization is as follows:

$$y_t = y_0 + rt + pt^2$$

where the parameter y_0 is the value of y_t when $t = 0$, r is the linear effect and represents the relative increase or decrease of y_0 for a unit increase of time t, and p is the deceleration of the growth parameter that suggests how much the exponential growth is slowing.

- The stationary point is $x_m = -\frac{r}{2p}$.
- The parameters have been estimated using an ordinary least squares (OLS) estimation (7).

1.3.3 Logistic regression, generalized logistic regression, and Richards regressions

The logistic regression curve models define an S-shaped growth with the Equation (8):

$$y_t = \frac{y_\infty}{1 + \left(\frac{y_\infty}{y_0} - 1 \right) e^{-rt}}$$

where y_∞ is the upper asymptote. The parameter r defines the logarithmic growth rate or steepness of the curve. Nonlinear least-square optimization was solved using the ML algorithm (5).

The generalized growth of logistic functions is an extension of logistic or sigmoid functions, allowing for more flexible S-shaped curves (9). The function is defined as (10) (11):

$$y_t = y_l + \frac{y_\infty - y_l}{(1+\exp(k(\log(t)-r)))^g}$$

where y_l is the lower asymptote, y_∞ is the upper asymptote, r is the growth rate, and k is the slope around the inflection point. The function is asymmetric for g different from l. Parameters are estimated using a general optimizer function to minimize the negative log-likelihood (12). The starting values to initialize the algorithm are defined considering a self-starting procedure as indicated in the literature (13).

Finally, the Richards model (RM) (9), also known as the power-law logistic model, is more flexible than the logistic one, since it has an additional parameter that controls the slowing rate of the exponential growth rate (14). The RM is described by the following equation (10,15):

$$y_t = y_l + \frac{y_\infty - y_l}{1+\exp(k(\log(t)-\log(r)))}$$

In the aforementioned equation y_t is the growth function at the time t, y_∞ is the upper asymptote, y_l is the lower asymptote, t is the time, k is the slope around the inflection point, and r is the growth rate, The nonlinear least-squares optimization was solved using the standard Levenberg-Marquardt (LM) algorithm (5).

1.3.4 Bertalanffy and Gompertz model

The Bertalanffy model is a growth model developed in the field of ecological research and is also used to describe the development of infectious diseases. The model is described by the following equation (10):

$$y_t = y_l + (y_\infty - y_l)\left(1-\exp\left(-\frac{t}{r}\right)\right)$$

In the equation y_t is the growth function at the time t, y_∞ is the upper asymptote of the growth function, y_l is the lower asymptote of the growth function, and r is the growth rate(16).
Nonlinear least-square optimization was solved using the standard LM algorithm (5).

The Gompertz model for a time series is a sigmoid function that describes a growth pattern that is slowest at both the start and the end of a given period. The right-hand or future-value asymptote of the function is approached much more gradually by the curve than the left-hand or lower-value asymptote. This goes against the simple logistic function in which both asymptotes are approached by the curve symmetrically(10) (15). Parameters are estimated using an optimizer function to minimize negative log-likelihood (12). The starting values to initialize the algorithm are defined considering a self-starting procedure as indicated in the literature (13).

1.3.5 Generalized additive model (GAM)

GAM (17) is a generalized model in which the linear predictor depends linearly on unknown smooth functions defined on the time variable. The GAM function is defined as

$y_t = y_0 + f_1(t)$

The f_1 is a function that may be defined considering a parametric, semiparametric, or not parametric smoothing. The scatterplot smoothing function with a natural spline was considered for computation. The optimization of the smoothness selection score has been carried out penalizing iteratively the reweighted least-squares scheme used to estimate the model given the smoothing parameters (18).

1.3.6 Poisson Generalized Linear Model (GLM).

A Poisson regression model may be defined considering the following parameterization.

$$\log(\lambda_t) = \log(N) + a + bt + ct^2 + dt^3$$

where N is the size of the population and λ_t is the parameter of the Poisson distribution of y_t. The estimation of model parameters is obtained using the ML approach performed using the Newton-Raphson (NR) algorithm (19).

1.3.7 ARIMA model

An Integrated Autoregressive Moving Average (ARIMA) model is a generalization of an Autoregressive Moving Average (ARMA) model, which is the basic model for analyzing stationary time series (20). In particular, the ARIMA models are used for non-stationary time series, where a differencing step can be applied to eliminate the non-stationarity.
Indicating with x the order of the autoregressive part, with y the degree of first differencing involved, and with z the order of the moving average part, let us model the epidemic series data y_t where t indicates the time, with an $ARIMA(x, y, z)$ model given by:

$$y'_t = c + \phi_1 y'_{t-1} + \cdots + \phi_x y'_{t-x} + \theta_1 \varepsilon_{t-1} + \cdots + \theta_z \varepsilon_{t-z} + \varepsilon_t$$

Where $\phi_i\ (i = 1, \ldots, x)$ are the autoregressive coefficients, and $\theta_i (i = 1, \ldots, z)$ are the moving average coefficients. The different epidemic series is y'_t. The predictors on the right side include lagged y values and error ε. The order of the non-seasonal ARIMA model was selected using the Bayesian information criterion (21).

1.3.8 Empirical Bayesian Time Series Framework

In the model proposed by Liu et al., the natural logarithm of the total cases is modeled by a functional effects model as follows:

$$y_t = y_0 + f(t) + \varepsilon_t$$

Where y_0 is the value of y_t when $t = 0$ and ε_t is the error component of the model. The functional effect (t_j) is modeled as a cubic smoothing spline with the state space representation as:

$$\begin{pmatrix} f(t) \\ f'(t) \end{pmatrix} = H_f \begin{pmatrix} f(t-1) \\ f'(t-1) \end{pmatrix} + \omega_{\mathrm{j}}$$

where $f'(t_j)$ is the first derivative concerning time, $H_f = \begin{pmatrix} 1 & \Delta t \\ 0 & 1 \end{pmatrix}$ is the state transition matrix, and Δt is the time interval between two points with the overall time range scaled to [0, 1], $\omega_j \sim Gaussian\left(0, \sum_f\right)$ is the state innovation vector, with

and α_f is a smoothing parameter.
Smoothing parameters and variances were estimated using the maximum penalized logarithmic likelihood approach (22):

$$\sum_f = \alpha_f^{-1} \begin{pmatrix} \frac{\Delta t^3}{3} & \frac{\Delta t^2}{2} \\ \frac{\Delta t^2}{2} & \Delta t \end{pmatrix}$$

All explanations of the model parameters are reported in Table 2.

References

1. Brauer F, Van Den Driessche P, Wu J. Mathematical Epidemiology. In: Mathematical Epidemiology [Internet]. 2008. p. 19–79. Disponibile su: https://doi.org/10.1007/978-3-540-78911-6_2

2. Lega J, Brown HE. Data-driven outbreak forecasting with a simple nonlinear growth model. Epidemics. dicembre 2016;17:19–26.

3. Cantó B, Coll C, Sánchez E. Estimation of parameters in a structured SIR model. Adv Differ Equ. 27 gennaio 2017;2017(1):33.

4. Malthus T, Gilbert G. An essay on the principle of population. Oxford university press. 2008. 172 p.

5. J.J. More. The Levenberg-Marquardt algorithm: implementation and theory,. Lecture Notes in Mathematics 630, Numerical Analysis. G.A. Watson (Ed.), Springer-Verlag. 1978;105–16.

6. Colman A. A dictionary of psychology. 3rd ed. Oxford University Press; 2009. 882 p.

7. Goldberger A. Econometric theory. New York Wiley; 1980. 399 p.

8. Vattay G. Predicting the ultimate outcome of the COVID-19 outbreak in Italy. 2020.

9. Richards F. J. A flexible growth function for empirical use. Journal of Experimental Botany. 1959;10(29):290–300.

10. Ritz C, Baty F, Streibig JC, Gerhard D. Dose-Response Analysis Using R. PLOS ONE [Internet]. 2015;10(e0146021). Disponibile su: http://journals.plos.org/plosone/article?id=10.1371/journal.pone.0146021

11. Finney DJ. Bioassay and the Practice of Statistical Inference. Int Stat Rev Rev Int Stat. 1979;47(1):1–12.

12. Ritz C, Streibig J. Bioassay Analysis Using R. J Stat Softw Artic. 2005;12(5):1–22.

13. Kniss AR, Vassios JD, Nissen SJ, Ritz C. Nonlinear Regression Analysis of Herbicide Absorption Studies. Weed Sci. 2017/01/20 ed. 2011;59(4):601–10.

14. Ma J. Estimating epidemic exponential growth rate and basic reproduction number. Infect Dis Model. 2020;5:129–41.

15. Seber GAF, Wild. Nonlinear Regression. New York: : Wiley \& Sons; 1989.

16. Jia L. Prediction and analysis of Coronavirus Disease 2019. 2019;(December).

17. Hastie T., Tibshirani R. Generalized additive models. Boca Raton, Fla: Chapman&Hall/CRC. 335 p.

18. Wood Simon N WSN. Thin plate regression splines. J R Stat Soc Ser B Stat Methodol. 28 gennaio 2003;65(1):95–114.

19. Cameron A, Trivedi P. Regression analysis of count data. Cambridge, UK ; New York, NY, USA: Cambridge University Press; 1998. 411 p.

20. Asteriou D, Hall S. Applied econometrics. 2nd ed. Basingstoke [England] ; New York: Palgrave Macmillan; 2011. 499 p.

21. Wit E, Heuvel E van den, Romeijn JW. 'All models are wrong...': an introduction to model uncertainty. Stat Neerlandica. 2012;66(3):217–36.

22. Liu Z, Guo W. Government Responses Matter: Predicting Covid-19 cases in US under an empirical Bayesian time series framework. medRxiv. 1 gennaio 2020;2020.03.28.20044578.

Table S1. Model parameters description.

**GENERAL**	
y_0	growth function at time *0*
y_t	growth function at time *t*
r	relative increase/decrease of y for a unit increase at time *t*
t	time (days)
**EXPONENTIAL**	
p	deceleration of growth that suggests how much the exponential growth is getting slower
**LOGISTIC, GENERALIZED LOGISTIC, AND RICHARDS REGRESSION**	
k	slope around the inflection point
g	Asymmetry parameter
**BERTANLAFFY AND GOMPERTZ MODEL**	
y_l	the lower asymptote of the growth function
k	slope around the inflection point
**GAM**	
$f_1(t)$	Smoothing function of time
y_0	growth function at time *0*
**POISSON GENERALIZED LINEAR MODEL**	
λ_t	parameter of the Poisson distribution
N	population size

**ARIMA**	
x	order of the autoregressive part
y	degree of first differencing involved
z	order of the moving average part
ϕ_i	autoregressive coefficient
θ_i	moving average coefficient
ε	error component
**EMPIRICAL BAYESIAN TIME-SERIES FRAMEWORK**	
y_0	growth function at time *0*
$f_{(tj)}$	functional effect
$e_{(ij)}$	Error component

Table S2. *MAE and MAPE for total cases.*

Panel A MAE

MAE Total cases

	20 days	30 days	40 days	50 days	60 days	70 days	80 days	90 days	98 days
**SIR**	41763.128	61631.441	35256.829	10347.885	6568666.971	31502.821	6156.542	5848.646	5864.783
**SIRD**	72337.880	58929.003	13365.466	11362.143	8943.640	9218.985	11300.318	14071.961	16238.300
**QUADRATIC**	97997.40	193751.40	154878.92	87321.94	47068.22	26310.69	15939.50	11814.39	10717.30
**POISSON**	10304600405	18490478.84	7220985.65	1001760.33	296970.06	126129.66	66763.88	44118.67	37380.12
**EXPONENTIAL**	3135047236.35	47584391.83	2155106.81	415599.38	152029.57	74771.32	45701.66	35436.41	33583.14
**LOGISTIC**	84445.80	50591.834	37550.557	24113.639	14249.145	8908.800	6675.314	5745.402	5490.985
**GOMPERTZ**	26425.484	76467.629	10019.812	10273.975	5271.497	2478.100	1730.360	1472.068	1375.564
**GENLOGIS**	68807.332	70585.583	300244.080	7279.645	1475.810	2471.948	1652.706	1220.359	1141.014
**BERTANLAFFY**	89307.25	40508.43	17808.07	21585.43	23136.68	21610.55	18999.54	13133.76	12356.63
**RICHARDS**	167294.370	57043.699	18413.441	10916.806	4130.433	1301.032	1303.410	1273.783	1251.391
**GAM**	28797.356	62634.039	39824.904	18572.346	10623.839	6455.077	2994.290	2432.309	2835.908
**ARIMA**	18794.23	46566.41	33809.95	13386.55	9144.54	2101.59	687.37	414.46	348.88

BAYESIAN	23598.51	54401.91	34764.97	14652.44	25118.037	1637.306	890.288	390.443	279.796

Panel B MAPE

MAPE Total cases

	20 days	30 days	40 days	50 days	60 days	70 days	80 days	90 days	98 days
SIR	0.261	0.349	0.269	0.216	27.863	0.303	0.210	0.214	0.215
SIRD	0.406	0.417	0.193	0.170	0.150	0.152	0.165	0.182	0.195
QUADRATIC	0.555	1.195	0.8488	1.312	2.114	2.997	3.893	4.645	5.091
POISSON	44665.04	806.048	32.2841	5.517	3.170	3.335	4.120	5.143	6.037
EXPONENTIAL	13605.50	208.49	10.55	3.92	3.99	5.00	6.309	7.696	8.798
LOGISTIC	0.477	0.315	0.300	0.394	0.581	0.790	0.951	1.075	1.156
GOMPERTZ	0.153	0.401	0.130	0.128	0.081	0.051	0.044	0.044	0.046
GENLOGIS	0.387	0.411	0.208	0.157	0.074	0.112	0.150	0.101	0.070
BERTANLAFFY	0.895	1.756	3.010	3.799	4.011	3.731	3.014	4.375	4.795
RICHARDS	0.839	0.347	0.210	0.116	0.089	0.176	0.206	0.197	0.183
GAM	0.194	0.303	0.218	0.187	0.287	0.181	0.1556	0.512	0.837
ARIMA	0.128	0.227	0.167	0.072	0.053	0.022	0.016	0.0.015	0.015
BAYESIAN	0.169	0.265	0.169	0.077	3.515	0.017	0.0134	0.011	0.011

Table S3. *MAE and MAPE for new cases.*

Panel A MAE

MAE New cases

	20 days	30 days	40 days	50 days	60 days	70 days	80 days	90 days	98 days
SIR	1975.778	2140.135	2156.117	2536.731	2488.577	2258.779	2025.271	1996.667	2001.496
SIRD	1627.866	1554.963	1202.559	923.431	646.679	469.803	480.777	528.935	531.310
QUADRATIC	30910	19248	1360	3125	2610	1813	1247	942.8	890.5
POISSON	138557124	1618152	45810	8243	3408	2086	1627	1481	1444
EXPONENTIAL	100371543	392235	18241	5178	2744	1937	1622	1508	1467
LOGISTIC	17123	2830	2153	1839	1628	1466	1626	1044	1040
GOMPERTZ	26256	3887	2221	1865	1647	1482	1626	1090	1090
GENLOGIS	26709	2317	2100	1825	1614	1445	1339	1292	1288
BERTANLAFFY	5132	7630	3690	2194	1790	1595	1477	1418	1398
RICHARDS	34816	3234	2173	1845	1631	1469	1364	1309	1553
GAM	13561	5993	3767	380.8	1008	581.8	441.8	434.6	429.3
ARIMA	5777	6249	1728	1046	865.7	471.8	380.8	357.9	350.2
BAYESIAN	8156	8185	1643	696	342.2	483.1	284.3	273.5	273.9

Panel B MAPE

MAPE New cases

	20 days	30 days	40 days	50 days	60 days	70 days	80 days	90 days	98 days
SIR	0.8637	1.359	1.390	2.194	2.100	1.626	1.073	0.979	0.998
SIRD	0.750	0.734	0.667	0.606	0.474	0.292	0.304	0.376	0.374
QUADRATIC	38.142	3.931	1.299	4.962	4.175	2.859	1.757	1.076	0.936
POISSON	286791.803	3040.128	72.106	11.602	4.732	3.017	2.466	2.325	2.316

**EXPONENTIAL**	206712.44	692.865	26.593	7.207	3.936	2.890	2.465	2.295	2.229
**LOGISTIC**	15.093	2.938	2.367	2.081	1.846	1.622	2.465	1.873	1.864
**GOMPERTZ**	27.880	3.931	2.460	2.125	1.880	1.651	2.465	1.920	1.920
**GENLOGIS**	26.508	2.551	2.315	2.051	1.804	1.574	1.368	1.218	1.161
**BERTANLAFFY**	6.035	8.895	4.198	2.620	2.188	1.912	1.671	1.480	1.357
**RICHARDS**	38.8	3.349	2.445	2.146	1.907	1.682	1.459	1.285	2.017
**GAM**	15.115	6.762	72.106	0.303	1.226	0.666	0.485	0.530	0.547
**ARIMA**	6.719	7.204	1.977	1.245	1.007	0.449	0.2771	0.2257	0.2046
**BAYESIAN**	9.372	9.247	2.05	0.7106	0.2401	0.5482	0.1737	0.1566	0.1552

Table S4. *Last observed vs last fitted total cases at i-th time.*

	**20 days**	**30 days**	**40 days**	**50 days**	**60 days**	**70 days**	**80 days**	**90 days**	**98 days**
**SIR (last observed)**	21157	69176	119827	159516	189973	210717	222104	229327	233019
**SIR (last fitted)**	21584.20	69267.20	122272.33	165011.35	193105.45	215604.48	228034.57	96197.26	96197.26
**SIRD (last observed)**	21157	69176	119827	159516	189973	210717	222104	229327	233019
**SIRD (last fitted)**	21369.23	72418.37	125415.32	164263.35	189484.98	203030.27	207872.75	332688.76	332931.49
**QUADRATIC (last observed)**	21157	69176	119827	159516	189973	210717	222104	229327	233019
**QUADRATIC (last fitted)**	19815	68149	127566	173080	206592	229415	240955	245054	245047
**POISSON (last observed)**	21157	69176	119827	159516	189973	210717	222104	229327	233019
**POISSON (last fitted)**	22617	81924	158469	219954	267407	302390	324637	337802	343866
**EXPONENTIAL (last observed)**	21157	69176	119827	159516	189973	210717	222104	229327	233019
**EXPONENTIAL (last fitted)**	21564	74769	137025	186574	226150	256095	275523	287865	294305
**LOGISTIC (last observed)**	21157	69176	119827	159516	189973	210717	222104	229327	233019
**LOGISTIC (last fitted)**	20941	69451	117652	152926	181000	201699	214170	221761	225759
**GOMPERTZ (last observed)**	21157	69176	119827	159516	189973	210717	222104	229327	233019
**GOMPERTZ (last fitted)**	20642	70390	120887	157701	186705	207951	220528	227936	231654
**GENLOGIS (last observed)**	21157	69176	119827	159516	189973	210717	222104	229327	233019
**GENLOGIS (last fitted)**	20747	69318	118942	157742	188518	211040	223924	231261	234889
**BERTANLAFFY (last observed)**	21157	69176	119827	159516	189973	210717	222104	229327	233019
**BERTANLAFFY (last fitted)**	15356	51490	102349	149617	190181	223356	248069	185229	203047
**RICHARDS REGRESSION (last observed)**	21157	69176	119827	159516	189973	210717	222104	229327	233019

**RICHARDS REGRESSION (last fitted)**	20735	70303	119861	157064	186899	208945	222047	229880	233887
**GAM (last observed)**	21157	69176	119827	159516	189973	210717	222104	229327	233019
**GAM (last fitted)**	20700	69934	120738	158167	189576	212539	224554	231311	235013
**ARIMA (last observed)**	21157	69176	119827	159516	189973	210717	222104	229327	233019
**ARIMA (last fitted)**	20207	68716	119910	160455	190697	211228	222618	384116	426108
**BAYESIAN (last observed)**	21157	69176	119827	159516	189973	210717	222104	229327	233019
**BAYESIAN (last fitted)**	2829	5984	4426	3713	2745	1580	908.2	606.2	443.2

Table S5. *Last observed vs last fitted new cases at the i-th time.*

	20 days	30 days	40 days	50 days	60 days	70 days	80 days	90 days	98 days
**SIR (last observed)**	3497	5249	4585	3153	2646	1389	888	669	355
**SIR (last fitted)**	3351.21	5275.58	3759.55	3106.70	1354.60	1910.79	1271.68	0	0
**SIRD (last observed)**	3497	5249	4585	3153	2646	1389	888	669	355
**SIRD (last fitted)**	3333.95	5154.26	3576.14	2613.25	2116.96	1548.51	1074.90	0	0
**QUADRATIC (last observed)**	3497	5249	4585	3153	2646	1389	888	669	355
**QUADRATIC (last fitted)**	3165	6359	5489	3716	2167	760.9	421.2	52795	62362
**POISSON (last observed)**	3497	5249	4585	3153	2646	1389	888	669	355
**POISSON (last fitted)**	3437	7666	7833	6564	5230	3954	2826	2023	1551
**EXPONENTIAL (last observed)**	3497	5249	4585	3153	2646	1389	888	669	355
**EXPONENTIAL (last fitted)**	3386	6731	6536	5585	4611	3659	2810	1625760	3379013
**LOGISTIC (last observed)**	3497	5249	4585	3153	2646	1389	888	669	355
**LOGISTIC (last fitted)**	3355	5791	5284	4729	4259	3799	2824	6383	6383
**GOMPERTZ (last observed)**	3497	5249	4585	3153	2646	1389	888	669	355
**GOMPERTZ (last fitted)**	3247	5925	5328	4724	4248	3789	2825	8179	8182
**GENLOGIS (last observed)**	3497	5249	4585	3153	2646	1389	888	669	355
**GENLOGIS (last fitted)**	3282	5588	5242	4750	4278	3804	3351	5588	5588
**BERTANLAFFY (last observed)**	3497	5249	4585	3153	2646	1389	888	669	355
**BERTANLAFFY (last fitted)**	2512	5519	5806	4924	4256	3719	3254	18807	20578
**RICHARDS REGRESSION (last observed)**	3497	5249	4585	3153	2646	1389	888	669	355
**RICHARDS REGRESSION (last fitted)**	3277	5817	5282	4719	4252	3796	3347	7092	7096
**GAM (last observed)**	3497	5249	4585	3153	2646	1389	888	669	355
**GAM (last fitted)**	3290	5730	4054	3637	3066	1990	1022	626.8	458.7
**ARIMA (last observed)**	3497	5249	4585	3153	2646	1389	888	669	416
**ARIMA (last fitted)**	2731	4962	4668	4092	3370	1900	1402	652	516
**BAYESIAN (last observed)**	3497	5249	4585	3153	2646	1389	888	669	355
**BAYESIAN (last fitted)**	2829	5984	4426	3713	2745	1580	908.2	606.2	443.2
